# Supplementary material for: Experiences of people living with HIV who have participated in psychological interventions: Protocol for a qualitative meta-analysis
Source: PLoS One. 2026 Mar 16;21(3):e0344798. doi: 10.1371/journal.pone.0344798 (PMC12991240; doi:10.1371/journal.pone.0344798)
Supplement: S1 Table — (PDF) [file pone.0344798.s001.pdf]

S1 Table. Full SPIDER-based search strategy for MEDLINE Complete (EBSCOhost).

Search date: Thursday, 19 February 2026. Interface limits applied: publication years January 2000–present; Humans; Peer-reviewed academic publications. Search mode: Proximity. SmartText and “Apply equivalent subjects” OFF for the final run. Because MEDLINE Complete limits each query line to ~255 characters, some concepts were entered across multiple sets and combined with OR.

SPIDER	Purpose	Set	Search strategy (MEDLINE via EBSCOhost)
**S (Sample)**	HIV infection / PLHIV (MeSH)	S1	MH "HIV Infections+"
**S (Sample)**	HIV terms in title	S2	TI (HIV OR AIDS OR PLHIV OR "people living with HIV" OR "living with HIV" OR HIV-positive OR "HIV positive" OR "HIV+" OR "human immunodeficiency virus" OR serostatus OR "sero status" OR "HIV seropositive" OR "HIV-seropositive")
**S (Sample)**	HIV terms in abstract	S3	AB (HIV OR AIDS OR PLHIV OR "people living with HIV" OR "living with HIV" OR HIV-positive OR "HIV positive" OR "HIV+" OR "human immunodeficiency virus" OR serostatus OR "sero status" OR "HIV seropositive" OR "HIV-seropositive")
**S (Sample)**	Sample combined	S4	S1 OR S2 OR S3
**P (Phenomenon of Interest)**	CMDs / common mental health problems (MeSH)	S5	MH ("Depression+" OR "Anxiety+" OR "Substance-Related Disorders+" OR "Suicidal Ideation")
**P (Phenomenon of Interest)**	CMDs / common mental health problems (text words)	S6	TX ("common mental disorder*" OR "common mental health problem*" OR depress* OR anxi* OR "psychological distress" OR (distress N2 psycholog*) OR "substance use" OR "drug use" OR alcohol* OR suicid* OR "self harm" OR "self-harm")
**P (Phenomenon of Interest)**	Phenomenon combined	S7	S5 OR S6
**D (Design)**	Psychotherapy (MeSH explode)	S8	MH "Psychotherapy+"
**D (Design)**	Core psychological intervention terms	S9	TX (psychotherap* OR "psychological intervention*" OR "psychological treatment*" OR "psychological counseling" OR CBT OR "cognitive therap*" OR "behavio?r* therap*" OR "interpersonal therap*" OR IPT)

**D (Design)**	Additional psychotherapy modalities	S10	TX ("supportive therap*" OR "group therap*" OR "group psychotherap*" OR "brief therap*" OR "problem-solving therap*" OR "problem solving therap*" OR "behavio?r* activation")
**D (Design)**	Mindfulness / ACT / DBT / EMDR (clean)	S11	TX ("mindfulness-based" OR "mindfulness based" OR MBSR OR MBCT OR "acceptance and commitment therap*" OR "dialectical behavio?r therap*" OR DBT OR EMDR OR "eye movement desensitization and reprocessing")
**D (Design)**	Psychodynamic / psychoanalytic	S12	TX (psychodynamic* OR psychoanal* OR "dynamic psychotherapy" OR "psychoanalytic psychotherapy" OR "psychodynamic psychotherapy" OR "short-term psychodynamic" OR "short term psychodynamic" OR "brief psychodynamic")
**D (Design)**	Psychoeducation/coping/self-management (anchored)	S13	TX (psychoeducat* OR ("coping" N3 (skill* OR strateg* OR intervention* OR program* OR training)) OR "coping skill*" OR ("stress management" N3 (intervention* OR program* OR training)) OR ("skills training" N3 (intervention* OR program*)) OR ("self-management" N3 (intervention* OR program* OR training)) OR (("behavio?r change") N3 (intervention* OR program* OR strategy OR counseling OR therap*)))
**D (Design)**	Interventions combined	S14	S8 OR S9 OR S10 OR S11 OR S12 OR S13
**R (Research type)**	Qualitative labels	S18	TI (qualitative OR "qualitative analys*" OR "qualitative study" OR "qualitative research") OR AB (qualitative OR "qualitative analys*" OR "qualitative study" OR "qualitative research")
**R (Research type)**	Interviews / focus groups	S19	TI (interview* OR "in-depth interview*" OR "in depth interview*" OR "focus group*" OR "semi-structured" OR semistructured) OR AB (interview* OR "in-depth interview*" OR "in depth interview*" OR "focus group*" OR "semi-structured" OR semistructured)
**R (Research type)**	Qualitative analysis approaches	S20	TI ("thematic analys*" OR "grounded theory" OR "content analys*" OR

			"framework analys*") OR AB ("thematic analys*" OR "grounded theory" OR "content analys*" OR "framework analys*")
**R (Research type)**	Ethnography / phenomenology	S21	TI (ethnograph* OR phenomenolog* OR "participant observation" OR "interpretative phenomenological") OR AB (ethnograph* OR phenomenolog* OR "participant observation" OR "interpretative phenomenological")
**R (Research type)**	Mixed methods / process evaluation (anchored)	S22	TI ("mixed method*" OR "mixed-method*" OR multimethod* OR "multi-method*" OR ("process evaluation" N3 (intervention* OR program* OR trial OR implement*))) OR AB ("mixed method*" OR "mixed-method*" OR multimethod* OR "multi-method*" OR ("process evaluation" N3 (intervention* OR program* OR trial OR implement*)))
**R (Research type)**	Research type combined	S23	S18 OR S19 OR S20 OR S21 OR (S22 AND (S18 OR S19 OR S20))
**E (Evaluation)**	Acceptability/experience **anchored to psychological interventions**	S24	TX ((acceptab* OR feasib* OR satisf* OR appropriateness OR usability OR "treatment preference*" OR "participant preference*" OR "patient preference*" OR "lived experience*" OR "user experience*" OR "patient experience*" OR "participant experience*" OR "treatment experience*" OR experience* OR perception* OR perspective* OR view* OR attitude*) N3 (psychotherap* OR "psychological intervention*" OR "psychological treatment*" OR CBT OR IPT OR MBCT OR MBSR OR DBT OR EMDR OR "psychodynamic psychotherapy" OR "dynamic psychotherapy"))
**Combine**	Final set (SPIDER order)	**S25**	**S4 AND S7 AND S14 AND S23 AND S24** [Final yield: n = 45]
