# Supplementary material for: Experiences of people living with HIV who have participated in psychological interventions: Protocol for a qualitative meta-analysis
Source: PLoS One. 2026 Mar 16;21(3):e0344798. doi: 10.1371/journal.pone.0344798 (PMC12991240; doi:10.1371/journal.pone.0344798)
Supplement: Attachment — Submitted filename: Response to Reviewers 11-02-26.docx [file pone.0344798.s004.docx]

**Response to Reviewers**

Manuscript ID: PONE-D-25-38173

Dear Dr. Mitra,

We sincerely thank you and the reviewer for the careful evaluation of our manuscript and for the positive and constructive feedback.

We are pleased that the reviewer considered the research question valid, the methodology technically sound, and the protocol sufficiently detailed and replicable.

Reviewer #1 noted a discrepancy between the time frame described in the abstract (10 years) and that described in the Methods section (25 years). We have carefully reviewed the manuscript and corrected this inconsistency. The abstract has been revised to specify that the review will include studies published between January 1, 2000 and February 23, 2026, consistent with the Methods section.

In addition, we have addressed all journal requirements outlined in the decision letter, including:

- Updating the Role of Funder statement to comply with PLOS ONE guidelines.
- Providing a complete Data Availability Statement in the submission form.
- Ensuring that the abstract in the manuscript and submission system are identical.
- Including the detailed MEDLINE (EBSCOhost) search strategy as S1 Table.
- Adding Supporting Information captions at the end of the manuscript.
- Reviewing and standardizing all references according to PLOS ONE formatting guidelines.

We believe that these revisions fully address the reviewer’s comment and the journal requirements. We thank you again for your consideration.

Sincerely,
